# Team-focused implementation strategies to improve implementation of mental health screening and referral in rural Children’s Advocacy Centers: study protocol for a pilot cluster randomized hybrid type 2 trial

**DOI:** 10.1186/s43058-023-00437-z

**Published:** 2023-05-26

**Authors:** Elizabeth A. McGuier, Gregory A. Aarons, Jaely D. Wright, John C. Fortney, Byron J. Powell, Scott D. Rothenberger, Laurie R. Weingart, Elizabeth Miller, David J. Kolko

**Affiliations:** 1 Department of Psychiatry, School of Medicine, University of Pittsburgh, 3811 O’Hara Street, Pittsburgh, PA 15213 USA; 2 Department of Psychiatry, University of California San Diego, La Jolla, CA USA; 3 UC San Diego ACTRI Dissemination and Implementation Science Center, La Jolla, CA USA; 4 Child and Adolescent Services Research Center, San Diego, CA USA; 5 Western Psychiatric Hospital, University of Pittsburgh Medical Center, Pittsburgh, USA; 6 Division of Population Health, Department of Psychiatry and Behavioral Sciences, University of Washington, Seattle, WA USA; 7 Department of Veterans Affairs, HSR&D Center of Innovation for Veteran-Centered and Value Driven Care, VA Puget Sound Health Care System, Seattle, WA USA; 8 Center for Mental Health Services Research, Brown School, Washington University in St. Louis, St. Louis, MO USA; 9 Division of Infectious Diseases, John T. Milliken Department of Medicine, Washington University School of Medicine, Washington University in St. Louis, St. Louis, MO USA; 10 Center for Dissemination and Implementation, Institute for Public Health, Washington University in St. Louis, St. Louis, USA; 11 Department of Medicine, University of Pittsburgh School of Medicine, Pittsburgh, PA USA; 12 Tepper School of Business, Carnegie Mellon University, Pittsburgh, PA USA; 13 Department of Pediatrics, University of Pittsburgh School of Medicine, Pittsburgh, PA USA

**Keywords:** Team, Teamwork, Implementation mapping, Implementation strategies, Mental health screening, Child maltreatment, Children’s Advocacy Centers

## Abstract

**Background:**

Children’s Advocacy Centers (CACs) use multidisciplinary teams to investigate and respond to maltreatment allegations. CACs play a critical role in connecting children with mental health needs to evidence-based mental health treatment, especially in low-resourced rural areas. Standardized mental health screening and referral protocols can improve CACs’ capacity to identify children with mental health needs and encourage treatment engagement. In the team-based context of CACs, teamwork quality is likely to influence implementation processes and outcomes. Implementation strategies that target teams and apply the science of team effectiveness may enhance implementation outcomes in team-based settings.

**Methods:**

We will use Implementation Mapping to develop team-focused implementation strategies to support the implementation of the Care Process Model for Pediatric Traumatic Stress (CPM-PTS), a standardized screening and referral protocol. Team-focused strategies will integrate activities from effective team development interventions. We will pilot team-focused implementation in a cluster-randomized hybrid type 2 effectiveness-implementation trial. Four rural CACs will implement the CPM-PTS after being randomized to either team-focused implementation (*n* = 2 CACs) or standard implementation (*n* = 2 CACs). We will assess the feasibility of team-focused implementation and explore between-group differences in hypothesized team-level mechanisms of change and implementation outcomes (implementation aim). We will use a within-group pre-post design to test the effectiveness of the CPM-PTS in increasing caregivers’ understanding of their child’s mental health needs and caregivers’ intentions to initiate mental health services (effectiveness aim).

**Conclusions:**

Targeting multidisciplinary teams is an innovative approach to improving implementation outcomes. This study will be one of the first to test team-focused implementation strategies that integrate effective team development interventions. Results will inform efforts to implement evidence-based practices in team-based service settings.

**Trial registration:**

Clinicaltrials.gov, NCT05679154. Registered on January 10, 2023.

**Supplementary Information:**

The online version contains supplementary material available at 10.1186/s43058-023-00437-z.

Contributions to the literature

- This study protocol describes the development and testing of team-focused implementation strategies to support mental health screening and referral in a team-based setting.
- This study will be among the first to develop team-focused strategies to enhance implementation.
- Findings from this study will inform efforts to implement new practices in team-based settings.

## Background

Child maltreatment and associated mental health problems are critical concerns, particularly in rural areas [1–4]. Children in rural areas are nearly twice as likely as their urban peers to experience child maltreatment [4], and they have high rates of unmet mental health needs [1, 2, 5, 6]. Maltreatment substantially increases the risk for mental health disorders [7–12]. Youth in rural areas are less likely to receive mental health care and experience greater impairment than those in urban areas [1, 2, 13, 14]. In addition, youth suicide rates in rural areas are nearly twice those in urban areas [5] and rising more rapidly [6]. Despite these high needs, implementation of evidence-based practices has lagged behind in rural areas [15–20].

Children’s Advocacy Centers (CACs) are intended to provide coordinated, interagency responses to maltreatment allegations and have wide reach into rural areas [21]. CACs are well-positioned to identify children at risk for mental health problems and suicide and to facilitate access to evidence-based treatments. There are approximately 1000 CACs in the USA, and more than 90% of children live in areas served by a CAC [22]. More than half of these CACs serve predominately rural populations [21, 23].

CACs are often families’ first link to services following maltreatment [24, 25]. Accreditation standards for CACs require “evidence-supported, trauma-focused” mental health services to be available to all children served by the CAC [26]. These services may be provided on-site (23.3% of CACs), through linkage agreements with local providers (33.8% of CACs), or through a combination of onsite services and linkage agreements (43%) [27]. Availability of evidence-based treatments (EBTs) through CACs has increased rapidly; in 2020, 98% of CACs reported they offer access to at least one EBT (100% of urban CACs; 98% of rural CACs) [28]. Trauma-Focused Cognitive-Behavioral Therapy (TF-CBT; [29]) is the most common EBT, offered by 94% of CACs [28].

Evidence-based screening tools can improve CACs’ capacity to identify children with mental health needs, and supported referrals (e.g., warm handoffs) can encourage treatment engagement. However, many CACs do not use evidence-based screening tools or standardized referral protocols [27]. Thirty-nine percent of CACs do not provide any on-site mental health screening [27], and referrals are typically provided by bachelor’s-level victim advocates with little specialized training in mental health. Implementation of structured screening and referral protocols can improve recognition of suicidality and mental health needs, reduce variability and inefficient use of resources, and facilitate engagement in treatment [30, 31﻿].

The Care Process Model for Pediatric Traumatic Stress (CPM-PTS) is a standardized mental health screening and referral protocol developed at the University of Utah with a grant from the Substance Abuse and Mental Health Services Administration (1U79SM080000) [32–35﻿]. Multidisciplinary stakeholders provided feedback to ensure fit of the CPM-PTS in the CAC context, including consideration of content, timing of administration, data storage, and legal protections. The CPM-PTS uses evidence-based tools to identify children with traumatic stress symptoms (UCLA PTSD Reaction Index Brief Form [﻿^36^]) and/or suicide risk (Columbia-Suicide Severity Rating Scale [﻿^37^]). It provides structured clinical pathways and technology-guided decision support to assist frontline CAC staff in understanding screening results, discussing mental health needs with youth and caregivers, and facilitating referrals to EBTs (e.g., TF-CBT [﻿^29^]).

**Fig. 1:**
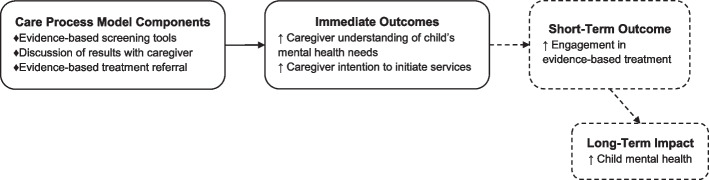
Hypothesized effects of the care process model for pediatric traumatic stress on family outcomes

The CPM-PTS is a promising approach to increasing engagement in evidence-based treatment for children at high risk for posttraumatic stress and other mental health problems. Care process models like the CPM-PTS aim to improve efficiency, increase accuracy, and decrease variability and have been shown to increase the provision of evidence-based care and reduce costs [38–41]. Electronic decision support tools have been shown to increase adherence to clinical guidelines and decrease cognitive load [42]. The core components of the CPM-PTS, including use of evidence-based screening tools, discussion of results with families, and referrals to evidence-based treatment, are hypothesized to increase engagement in mental health services by increasing caregivers’ understanding of their children’s mental health needs and their intentions to initiate services (Fig. 1). However, the effect of the CPM-PTS on family-level outcomes has not yet been tested. In addition, effective strategies for implementing mental health screening/referral protocols such as the CPM-PTS in the unique context of CACs are needed.

CAC accreditation standards require the use of a multidisciplinary team, including members from law enforcement, child welfare, prosecution, medicine, mental health, and victim advocacy [26]. Small rural CACs may have as few as one employee, relying primarily on team members employed by independent organizations (e.g., child welfare, mental health agencies). CACs lack a conventional hierarchical structure (e.g., frontline staff, mid-level managers, a central executive) and require cross-sector collaboration and effective teamwork to be successful [43]. In this context, the multidisciplinary team is likely to play a central role in the implementation of evidence-based practices. Our research applies the science of teams and team effectiveness to implementation and use of evidence-based practices in rural CACs in the USA.

**Fig. 2:**
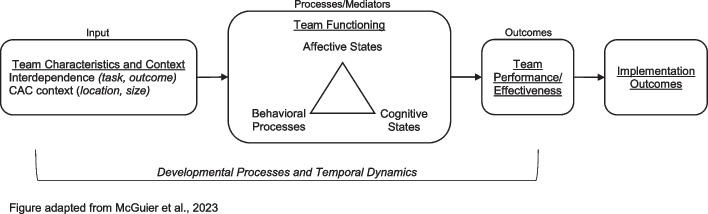
Input-mediator-outcome framework of team effectiveness

Figure 2 illustrates our conceptual model of team performance and implementation outcomes, based on the Input-Mediator-Outcome model [44–47]. We focus on team interdependence and functioning [48–50], as team structure and task demands are relatively constant across CACs because of national accreditation standards [26]. *Interdependence* is the extent to which the team’s work requires exchanges of resources and coordinated workflows (i.e., task interdependence) and the extent to which outcomes are measured and rewarded at the team (vs. individual) level (i.e., outcome interdependence) [48, 51]. *Team functioning* refers to processes (e.g., coordination) and emergent states (e.g., cohesion) that may be affective, behavioral, or cognitive [49, 50]. Within organizations, team interdependence and functioning are positively associated with team performance [48–55], and in healthcare settings, patient safety and clinical outcomes [56–58].

In CACs, qualitative research has identified clear interagency policies and procedures as key facilitators of cross-sector collaboration [59–61], and one study found that greater interdependence (i.e., more frequent case review meetings, use of a joint performance evaluation system) was associated with higher quality team relationships [62]. Our prior research with CAC multidisciplinary teams found that affective and cognitive functioning were positively associated with team performance [43]. But, little research has examined teams’ impact on implementation processes and outcomes [63, 64]. Some evidence suggests that problems with team functioning (e.g., low cohesion, ineffective communication, high conflict) impair implementation of new practices [65–69].

In prior research with CACs, we evaluated a statewide initiative to implement the CPM-PTS [32]. We found that affective functioning (i.e., trust, liking, and respect within the team) and team performance were associated with greater acceptability, appropriateness, and feasibility of the protocol [70]. Task interdependence was positively associated with reach, with teams with greater exchanges of resources and coordinated workflows achieving higher screening rates during the first two years of implementation [70]. Our findings suggest that strategies that improve team interdependence and functioning may enhance implementation outcomes in team-based settings.

## Current study

The current study is a hybrid type 2 effectiveness-implementation pilot cluster randomized trial in four rural Children’s Advocacy Centers. Hybrid type 2 studies give roughly equal emphasis to evaluating implementation strategies and intervention effectiveness [71, 72]. We will develop and pilot team-focused strategies to enhance the implementation of the CPM-PTS in CACs. The primary goal of the trial is to assess the feasibility of the implementation strategies and trial methods [73–77]. We will explore between-group differences in hypothesized team-level mechanisms of change and implementation outcomes (implementation aim). We will use a within-group pre-post design to test the effectiveness of the CPM-PTS in increasing caregivers’ understanding of their child’s mental health needs and caregivers’ intentions to initiate services (effectiveness aim).

### Study aims and hypotheses

Aim 1: Develop team-focused strategies to facilitate implementation in rural CACs.

Aim 2: Conduct a pilot cluster-randomized controlled effectiveness-implementation hybrid Type II trial in 4 rural CACs.

Aim 2a (implementation aim): Assess the feasibility of team-focused implementation strategies and explore between-group differences in team interdependence and functioning and implementation outcomes (i.e., days to adoption, reach, acceptability, appropriateness, feasibility).

*Hypothesis (primary)*: Team-focused implementation will be judged to be feasible, acceptable, and appropriate (scores ≥ 4 on 1-5 scale).

*Hypothesis (exploratory)*: Team interdependence and functioning will be greater in CACs randomized to team-focused implementation than comparison CACs.

*Hypothesis (exploratory)*: Implementation outcomes, including days to adoption (i.e., days from training to first use), reach (i.e., percent of children screened), and CPM-PTS acceptability, appropriateness, and feasibility, will be greater in CACs randomized to team-focused implementation than comparison CACs.

Aim 2b (effectiveness aim): Test the effect of CPM-PTS implementation on caregivers’ understanding of their child’s mental health needs and caregivers’ intentions to initiate mental health services.

*Hypothesis (primary):* Understanding of mental health needs and intentions to initiate mental health services will be greater for caregivers served after the CPM-PTS is implemented than caregivers served before the CPM-PTS is implemented.

*Hypothesis (exploratory):* Referrals to mental health services and initiation of mental health services will be greater for children served after the CPM-PTS is implemented than children served before the CPM-PTS is implemented.

### Community engagement

A community-engaged approach with bi-directional involvement of researchers and community stakeholders will be used throughout the study [78–81]. A community advisory committee of multidisciplinary team members and CAC leadership will provide feedback on research questions, study methods, interpretation of results, and dissemination plans. The committee will meet via videoconferencing at least once a quarter, and engagement processes and proximal outcomes will be assessed following best practice recommendations [81–85]. For example, we will assess the frequency and duration of engagement, committee members’ experiences of decision-making, and changes in project methods (e.g., methods, recruitment) and interpretation of results based on committee input [82, 83]. Committee members may change during the study; we will strive to maintain diverse representation from the disciplines involved in CACs and individuals from rural and urban areas.

## Aim 1 methods

### Development of team-focused implementation strategies

We will use Implementation Mapping to develop and refine implementation strategies. Implementation Mapping is a systematic, participatory, theory-based process based on Intervention Mapping [86–92]. Table 1 lists the five steps in the process identified by Fernandez and colleagues [89].

**Table 1:** Implementation mapping steps [89]

**Step**	**Task(s)**
1	Conduct a needs assessment and identify adopters and implementers.
2	Identify adoption and implementation outcomes, performance objectives, and determinants; create matrices of change.
3	Choose theoretical methods; select or create implementation strategies.
4	Produce implementation protocols and materials.
5	Evaluate implementation outcomes.

Step 1 will be completed in collaboration with our community advisory committee. The committee will begin by reviewing findings from our evaluation of the statewide CPM-PTS implementation effort [﻿^32^]. They will identify individuals likely to be responsible for adopting, using, and sustaining the CPM-PTS and generate additional relevant determinants.

Steps 2 and 3 will be completed through close collaboration of the research team and a subset of committee members. First, we will refine the study’s conceptual model and identify key team-related determinants (e.g., interdependence, supportive behavior). This step will include reviewing recent developments in the scientific literature and will be informed by our prior research on how specific types of team interdependence and functioning relate to implementation outcomes [70]. The research team will present summaries of relevant research for discussion and create initial drafts that committee members will review and revise during recurring meetings over a 3-month period.

The development of team-focused strategies will be informed by research on team development interventions. We will focus on two well-established types of team development interventions—team training and team building [93–98]. Team training targets team members’ knowledge, skills, and attitudes through strategies such as team self-correction, coordination and adaptation training, and cross-training and is effective in improving affective, behavioral, and cognitive team functioning [56, 93, 98–101]. Team building targets goal-setting, relationship management, role clarification, and/or problem-solving and is effective in improving team processes (e.g., coordination) and affective outcomes (e.g., cohesion) [94, 102].

We will design practical implementation strategies using strategies generated by committee members and adapted from existing team development interventions (e.g., TeamSTEPPS [99, 103–106]). Strategies will incorporate effective training methods (e.g., role play, feedback) and follow evidence-based recommendations for team interventions [95–98]. Table 2 presents examples of intervention targets, evidence-based intervention strategies, and practical examples of activities.

**Table 2:** Examples of team-level targets, strategies, and activities

**Intervention target**	**Strategy**	**Activities**
Outcome interdependence	Goal-setting	Team goal-setting exercise Feedback on team progress toward goal
Supportive/backup behavior	Cross-training	Train additional team members in CPM-PTS Role play CPM-PTS administration
Learning behavior	Self-correction	Education on communication skills Debriefing exercises

Step 4, creation of implementation protocols and materials, will be completed by the research team. We will operationalize team strategies (e.g., determining sequence, delivery method) and produce materials to support their use. Examples include a team intervention manual, scripts and worksheets for specific team activities (e.g., goal setting exercise, role play debriefing), and templates for protocol documents and interagency agreements. The community advisory committee will provide feedback on drafts and will be encouraged to test materials with their own teams to obtain additional feedback. Materials are intended to have potential for broad dissemination into low-resource settings providing team-based care, although some are specific to CACs.

Our final team-focused implementation plan will integrate team-level strategies with standard implementation strategies based on the Replicating Effective Programs (REP) model [107]. REP is a low-intensity approach to implementation that focuses primarily on the development and provision of an intervention package or toolkit, provider training, and technical assistance [108, 109]. Team-focused strategies will be integrated with standard implementation strategies provided to all sites (i.e., toolkit, training, technical assistance). For example, education on communication skills (team training activity) could be integrated with CPM-PTS training (REP strategy), and feedback on team progress (goal-setting activity) could be integrated with technical assistance calls (REP strategy). To flexibly adjust to variations in team needs, we will incorporate opportunities for CACs to choose specific activities.

Step 5 will be completed by the research team in collaboration with the committee. Committee members will review and refine the evaluation plan proposed by the research team to ensure its appropriateness in the setting. The evaluation will include an assessment of team-level determinants hypothesized to be mechanisms of change for team-focused implementation strategies.

## Aim 2 methods

### Study design

The pilot trial is a cluster-randomized hybrid type 2 effectiveness-implementation study. It is designed to evaluate the feasibility of team-focused implementation and explore differences in team and implementation outcomes (Aim 2a—implementation) as well as test the effectiveness of the CPM-PTS (Aim 2b—effectiveness). Four rural CACs will implement the CPM-PTS after being randomized to either team-focused implementation (*n* = 2 CACs) or standard implementation (*n* = 2 CACs). Supplemental File 1 includes the SPIRIT checklist [110, 111], StaRI checklist [112, 113], and CONSORT checklist and flow diagram [114] for this protocol paper. The trial is registered on clinicaltrials.gov (NCT05679154).

### Site recruitment and randomization

CACs (*N* = 4) will be recruited through the Pennsylvania Chapter of CACs, which provides training, support, and technical assistance to CACs, as well as direct outreach to CAC staff. Eligible CACs must be interested in implementing a mental health screening and referral protocol and in a county designated as rural by the Center for Rural Pennsylvania. CACs with members that participated in Aim 1 will not be eligible for the pilot trial. After completion of baseline data collection, CACs will be randomized to standard implementation (*n* = 2) or team-focused implementation (*n* = 2). We will aim to balance team size across conditions by creating pairs of CACs with similarly sized teams that are then randomized to condition by the study statistician using a random number generator. CACs will be informed of their study condition after baseline data collection is completed.

### Methods for Aim 2a (implementation aim)

#### Participants

All members of multidisciplinary teams at participating CACs will be invited to participate (estimated *N* = 70 [25 team members per CAC; 70% participation]). We expect team members to change over time and will include only current team members at each timepoint (0, 6, and 12 months), as individuals no longer on the team will not be able to accurately report on team functioning. We will work with our community advisory committee to develop effective recruitment and retention strategies.

#### Implementation conditions

CACs randomized to standard implementation (*n* = 2) will receive CPM-PTS implementation strategies based on the REP model and used in the Utah statewide implementation. They will receive a toolkit of CPM-PTS materials (e.g., manual, REDCap surveys, referral protocols), a short interactive training, and 6 months of technical assistance. CACs randomized to team-focused implementation (*n* = 2) will follow the plan developed in Aim 1 that integrates team strategies with standard training and technical assistance strategies, delivered over 6 months.

#### Team data collection procedures

Data will be collected through online surveys of team members at 0, 6, and 12 months. Consent forms and surveys will be constructed in REDCap, a secure, web-based software platform [115, 116], and individual survey invitations will be emailed to all team members at each timepoint. We will also conduct semi-structured qualitative interviews assessing team functioning with two team members from each CAC at baseline, 6 months, and 12 months. Interviews are intended to complement quantitative survey data by providing opportunities for elaboration and greater depth of understanding of team functioning. Interviews will be conducted via videoconference, audio-recorded, and transcribed.

Ethnographically informed “periodic reflections” on the implementation process [117] will be conducted approximately monthly during the 6 months of implementation support. Reflections will be conducted via videoconference and audio-recorded; the interviewer will take notes and summarize each interview immediately after it is completed. Participants will be paid for completing surveys and/or interviews. All procedures are approved by the University of Pittsburgh Institutional Review Board.

#### Measures

##### Feasibility

The primary goal of the trial is to assess the feasibility of the trial methods [73–77]. Accordingly, we will track site recruitment and retention, assess CAC characteristics that may affect implementation outcomes (e.g., team size, co-location, budget), and track team turnover, survey response rates, and missing data. Periodic reflections with key informants in each CAC (e.g., director, coordinator) will provide detailed information on the implementation process as it occurs. These structured reflections will include questions about implementation progress and completion of specific activities, barriers and facilitators to implementation, feedback on implementation strategies, and events and external influences that may impact implementation (e.g., leadership changes, new policies) [118–120]. Team members in CACs randomized to team-focused implementation will also rate the acceptability, appropriateness, and feasibility of team-focused implementation [121].

##### Team outcomes

At 0, 6, and 12 months, team members will complete an online survey assessing team interdependence, functioning, and performance. Measures are listed in Table 3. We will also assess other relevant determinants (e.g., leadership, resources, individual characteristics) [122–125]. Changes to measures may be made prior to the start of the trial to ensure an effective assessment of the hypothesized mechanisms of change for the team-focused implementation strategies developed in the Aim 1 Implementation Mapping process.

**Table 3:** Team member survey measures

**Domain**	**Construct**	**Number of items, rating scale**
Team Member Characteristics	Age, race/ethnicity, gender Discipline, experience, tenure	7 items
Team Interdependence	Task interdependence [51]	5 items, 5-point Likert scale
	Outcome interdependence [51]	2 items, 5-point Likert scale
Affective Team Functioning	Liking [126]	4 items, 5-point Likert scale
	Psychological safety [127]	7 items, 7-point Likert scale
	Team roles and respect [126, 128]	5 items, 5-point Likert scale
Behavioral Team Functioning	Learning behavior [127]	7 items, 7-point Likert scale
	Supportive behavior [129]	4 items, 5-point Likert scale
	Communication – information exchange [130]	4 items, 5-point Likert scale
	Relational coordination [131]	7 items, 5-point Likert scale
	Conflict management [132]	5 items, 5-point Likert scale
	Reflexivity [133]	4 items, 5-point Likert scale
Cognitive Team Functioning	Clear direction [127]	3 items, 7-point Likert scale
	Shared awareness [134, 135]	6 items, 5-point Likert scale
Team Performance	Team member-rated performance [127]	5 items, 7-point Likert scale
Implementation Determinants	Implementation climate [122]	4 items, 5-point Likert scale
	Leadership engagement [122]	4 items, 5-point Likert scale
	Available resources [122]	7 items, 5-point Likert scale
Implementation Outcomes	Acceptability, appropriateness, and feasibility [121] -Of CPM-PTS -Of team-focused implementation	12 items, 5-point Likert scale

##### Implementation outcomes

Adoption and reach will be assessed with data from CPM-PTS electronic administration and CAC administrative data. Data are collected in REDCap as the CPM-PTS is administered; we will use timestamps to determine the date of the first completed screening and assess the number of completed screenings each month. CACs will provide data on the number of children served each month. Adoption will be indicated by the number of days from training to the first completed screening. Reach will be indicated by screening rates (i.e., completed screenings/eligible children) and calculated for monthly and quarterly periods. Team members will rate the acceptability, appropriateness, and feasibility of the CPM-PTS at 0, 6, and 12 months [121].

#### Statistical analyses

##### Feasibility

We will examine descriptive statistics (e.g., mean, median, range) for quantitative measures of feasibility, acceptability, and appropriateness [121]. We expect mean scores to indicate agreement that team-focused implementation is feasible, acceptable, and appropriate (i.e., scores ≥ 4 on 1–5 scale; primary hypothesis). We will evaluate data completeness and quality and look for patterns of missing data.

##### Quantitative analyses

This study is not powered to detect between-group differences. We will explore differences in team outcomes (i.e., interdependence, functioning, performance) and implementation outcomes at 6 and 12 months (exploratory hypotheses). For team outcomes, we will construct mixed effects models to estimate effect sizes and confidence intervals. We will create separate estimates and confidence intervals for each condition (i.e., team-focused implementation vs. standard implementation). Analyses will not account for the matched pairing of CACs given the low number of clusters. For implementation outcomes, we will aggregate scores for outcomes rated by team members and examine descriptive statistics (e.g., mean, range) across CACs.

##### Qualitative analyses

We will conduct thematic analysis of team member interviews using a primarily theoretical (deductive) approach [﻿^136^]. A preliminary codebook will include a priori codes for specific dimensions of team functioning (e.g., psychological safety, supportive behavior, conflict management) and implementation determinants (e.g., implementation climate, knowledge, and beliefs). Two coders will read all transcripts and refine and add codes as needed through an iterative analysis process. After finalizing the codebook, all transcripts will be independently coded by two coders and discrepancies will be resolved through consensus.

We will conduct rapid analysis of periodic reflections [﻿^117^, ^137^, ^138^]. Interviewers will take detailed notes and summarize each reflection using a spreadsheet template. The template will list multiple domains (e.g., implementation progress, challenges, suggestions for change) and provide space for key points and exemplar quotes in each domain. To enhance validity, we will ask each CAC to provide feedback on findings (i.e., member checking) [139–141].

##### Mixed methods analyses

We will integrate survey and interview data on team functioning to examine triangulation (i.e., compare results from each method; function: convergence) and elaborate on quantitative findings (i.e., deepen understanding; function: complementarity) [142, 143]. Quantitative data on implementation will be used to assess outcomes, and qualitative data from periodic reflections will be used to understand process (function: complementarity). Qualitative findings will also be used to explain quantitative findings and explore any unexpected findings (function: expansion) [142, 143]. We will integrate quantitative and qualitative data on implementation to refine team-focused implementation strategies. For example, if we identify implementation activities with low completion, we will use qualitative data to identify barriers and suggestions for improving these activities.

### Methods for Aim 2b (effectiveness aim)

#### Participants and procedures

Anonymized caregiver data will be collected continuously through an existing Outcome Measurement System [144] over an 18-month period. No caregivers or children will be enrolled in the study. At the end of their CAC visit, caregivers complete a brief anonymous survey assessing satisfaction with their experience. We will obtain post-visit survey data for caregivers served in the 6 months preceding CPM-PTS implementation and data for caregivers served in the 12 months following CPM-PTS implementation. We estimate a total sample of 288 caregivers (4 CACs*10 caregivers/month*40% response rate*18 months). We will also collect administrative data from CAC case management systems documenting referrals to mental health services and when available, data on initiation of mental health services for cases served during the 18-month study period.

#### Measures

In the post-visit survey, caregivers will rate two items assessing their understanding of their child’s mental health needs and their intention to initiate mental health services. For each child served during the study period, we will extract two dichotomous (yes/no) variables from administrative data to indicate (1) if the child was referred to mental health services and (2) if the child initiated mental health services.

#### Statistical analyses

First, we will examine data completeness and patterns of missing data for caregiver survey ratings and CAC administrative data on referrals and initiation of mental health services. We will test the effect of CPM-PTS implementation on caregivers’ understanding of mental health needs and intentions to initiate services (primary hypothesis) using multilevel linear regression models. Models will use caregiver data from all sites and include a fixed effect of pre- vs. post-implementation and a random effect of CAC to account for clustering. If model convergence becomes a problem, we will apply robust standard errors to adjust for clustering. We will also explore outcomes using an interrupted time series regression model [145, 146]. Because differences in implementation between CACs may affect our estimates of CPM-PTS effectiveness, we may conduct exploratory “dosage adjusted” analyses using weighted regression models with weights proportional to screening rates to account for differences in use of the CPM-PTS between CACs. Lastly, if there are sufficient data, we will explore changes in mental health referrals and treatment initiation after CPM-PTS implementation (exploratory hypothesis). For each CAC, we will calculate the proportion of children referred to mental health services and the proportion initiating treatment during the 6 months preceding CPM-PTS implementation and the 12 months following CPM-PTS implementation and look for changes from pre-implementation to post-implementation.

Power consideration for our primary effectiveness hypothesis (i.e., changes in caregivers’ understanding of mental health needs and intentions to initiate services) is based on testing regression coefficients in multilevel models. To detect a standardized effect size of Cohen’s *d* = 0.35 from pre-implementation to post-implementation using a two-sided *t*-test at the 5% significance level with 80% power, a “standard design” with no clustering would require 292 caregivers. We multiplied this sample size by the design effect (DE) to account for clustering. The DE is equal to (1-ICC) because the implementation predictor has zero between-cluster variation, making the multilevel design more efficient than the standard design for this aim. Incorporating the DE reduces the required sample sizes to 278 and 263 for ICCs of 0.05 and 0.10, respectively. With our estimated sample size of 288, we will achieve at least 80% power to detect small-to-medium effects (*d* = 0.35) for a range of ICCs.

### Data and safety monitoring

The principal investigator will hold primary responsibility for monitoring the safety of this trial. The trial involves a non-pharmacological intervention provided to adult team members and the risk for serious adverse events is low; therefore, a Data and Safety Monitoring Board will not be appointed for this study. We will report any serious and unexpected adverse events to the Institutional Review Board (IRB) in accordance with IRB policy. The research team will meet regularly to discuss administrative issues and raise any concerns, and mentorship team meetings will include review and discussion of participant safety and privacy and the integrity, validity, and confidentiality of data collection and analyses. All participant information and data will be stored on a secure server. Any changes to study procedures will be approved by the IRB and reported in an update to the registered trial protocol. Members of the mentorship team can access the data by request after obtaining IRB approval.

### Dissemination plans

Study findings will be disseminated locally and nationally through multiple means, including (1) presentations to the community advisory committee; (2) presentations to participating CACs and CAC-related organizations, such as the Pennsylvania Chapter of CACs; (3) presentations at scientific and practice-oriented conferences; and (4) peer-reviewed journal articles. We will use the International Committee of Medical Journal Editors [147] criteria to make authorship decisions. The community advisory committee will be actively involved in determining strategies for disseminating results, particularly to CACs and associated stakeholder groups (e.g., leadership, team members, caregivers). This will help ensure that study findings and their implications can be immediately communicated to support practice initiatives and guide subsequent research investigations.

## Discussion

This study is innovative in its focus on CACs, a non-traditional setting, in rural areas. Extending the reach of evidence-based practices to the 13.4 million American children living in rural areas is crucial to public health impact. Effective screening and referral protocols can increase accurate identification of mental health needs, facilitate access to care, maximize efficient use of limited resources, and ultimately reduce rural disparities in mental health care. We will test the effectiveness of the CPM-PTS in increasing caregivers’ understanding of mental health needs and intentions to initiate services and explore its effectiveness in increasing treatment referrals and treatment initiation for children served by CACs.

Our team-focused implementation strategies reflect an innovative approach to improving implementation outcomes and are aligned with broader movements toward team-based care. We will use a rigorous Implementation Mapping process to develop team strategies and will adapt strategies proven to improve functioning in business and acute healthcare settings to non-acute settings. Consistent with calls to examine mechanisms in implementation science [148–150], we will assess possible team-level mechanisms of change for these strategies. Although this pilot trial is not powered to detect group differences in team and implementation outcomes, it will provide important feasibility data to support a future fully powered trial. The team strategies developed in this study may be generalizable to other teams with limited resources providing care across organizational and disciplinary boundaries and relying on cross-sector collaboration. Understanding how multidisciplinary teams affect the implementation process, and subsequently developing team-focused strategies to enhance implementation, can advance efforts to deliver evidence-based practices in team-based service settings.

## Supplementary Information

**Additional file 1.** SPIRIT 2013 Checklist, StaRI Checklist, CONSORT 2010 Checklist, CONSORT Flow Diagram.

## Data Availability

Data sharing is not applicable to this article as no datasets were generated or analyzed during the current study.

## References

[1] Anderson NJ, Neuwirth SJ, Lenardson JD, Hartley D (2013). Patterns of care for rural and urban children with mental health problems.

[2] Gamm LD, Hutchinson LL, Dabney BJ, Dorsey AM, editors. Rural Healthy People 2010: A companion document to Healthy People 2010. Vols. 1–3. College Station, TX: The Texas A&M University System Health Science Center, School of Rural Public Health, Southwest Rural Health Research Center; 2003 [Cited 2015 Jun 10]. Available from: http://sph.tamhsc.edu/centers/rhp2010/litreview/Vol3Ch1LR.htm.

[3] Maguire-Jack K, Jespersen B, Korbin JE, Spilsbury JC (2021). Rural child maltreatment: a scoping literature review. Trauma Viol Abuse..

[4] Sedlak AJ, Mettenburg J, Basena M, Peta I, McPherson K, Greene A (2010). Fourth national incidence study of child abuse and neglect (NIS-4).

[5] Fontanella CA, Hiance-Steelesmith DL, Phillips GS, Bridge JA, Lester N, Sweeney HA (2015). Widening rural-urban disparities in youth suicides, United States, 1996–2010. JAMA Pediatr.

[6] Ivey-Stephenson AZ, Crosby AE, Jack SPD, Haileyesus T, Kresnow-Sedacca MJO (2017). Suicide trends among and within urbanization levels by sex, race/ethnicity, age group, and mechanism of death — United States, 2001–2015. MMWR Surveill Summ.

[7] Fitzgerald MM, Berliner L. Psychosocial consequences and treatments for maltreated children. In: Korbin JE, Krugman RD, editors. Handbook of Child Maltreatment. Springer Netherlands; 2014 [Cited 2014 Apr 9]. 377–92. (Child Maltreatment). Available from: http://link.springer.com/chapter/10.1007/978-94-007-7208-3_20 .

[8] Halpern SC, Schuch FB, Scherer JN, Sordi AO, Pachado M, Dalbosco C (2018). Child maltreatment and illicit substance abuse: a systematic review and meta-analysis of longitudinal studies. Child Abuse Rev.

[9] Jaffee SR (2017). Child maltreatment and risk for psychopathology in childhood and adulthood. Ann Rev Clin Psychol.

[10] Su Y, D’Arcy C, Meng X (2022). Intergenerational effect of maternal childhood maltreatment on next generation’s vulnerability to psychopathology: a systematic review with meta-analysis. Trauma Viol Abuse.

[11] Vizard E, Gray J, Bentovim A (2022). The impact of child maltreatment on the mental and physical health of child victims: a review of the evidence. BJPsych Adv.

[12] Widom CS. Longterm consequences of child maltreatment. In: Korbin JE, Krugman RD, editors. Handbook of Child Maltreatment. Springer Netherlands; 2014 [cited 2014 Apr 9]. p. 225–47. (Child Maltreatment). Available from: http://link.springer.com/chapter/10.1007/978-94-007-7208-3_12.

[13] Smalley KB, Yancey CT, Warren JC, Naufel K, Ryan R, Pugh JL (2010). Rural mental health and psychological treatment: a review for practitioners. J Clin Psychol.

[14] Wang P, Lane M, Olfson M, Pincus H, Wells W, Kessler RC (2005). Twelve-month use of mental health services in the united states: results from the national comorbidity survey replication. Arch Gen Psychiatry.

[15] Parsons JE, Merlin TL, Taylor JE, Wilkinson D, Hiller JE (2003). Evidence-based practice in rural and remote clinical practice: where is the evidence?. Australian J Rural Health.

[16] Dotson JAW, Roll JM, Packer RR, Lewis JM, McPherson S, Howell D (2014). Urban and rural utilization of evidence-based practices for substance use and mental health disorders. J Rural Health.

[17] Smith TA, Adimu TF, Martinez AP, Minyard K (2016). Selecting, adapting, and implementing evidence-based interventions in rural settings: an analysis of 70 community examples. J Health Care Poor Underserved.

[18] Louison L, Fleming O (2016). Context matters: Recommendations for funders & program developers supporting implementation in rural communities.

[19] Palinkas LA, Holloway IW, Rice E, Fuentes D, Wu Q, Chamberlain P (2011). Social networks and implementation of evidence-based practices in public youth-serving systems: a mixed-methods study. Implement Sci.

[20] Boydell KM, Stasiulis E, Barwick M, Greenberg N, Pong R (2008). Challenges of knowledge translation in rural communities: the case of rural children’s mental health. Can J Commun Mental Health.

[21] National Children’s Alliance (2020). National Children’s Alliance - Annual Report 2019.

[22] National Children’s Alliance (2022). CAC Coverage Maps. National Children’s Alliance.

[23] National Children’s Alliance (2016). Snapshot 2017: Advocacy, efficacy, and funding in CACs.

[24] Elmquist J, Shorey RC, Febres J, Zapor H, Klostermann K, Schratter A (2015). A review of Children’s Advocacy Centers’ (CACs) response to cases of child maltreatment in the United States. Aggress Violent Behav.

[25] Herbert JL, Bromfield L (2019). Multi-disciplinary teams responding to child abuse: common features and assumptions. Children Youth Serv Rev.

[26] National Children’s Alliance (2017). Standards for accredited members - 2017 edition.

[27] National Children’s Alliance (2019). 2018 NCA Member Census Report - Mental Health Section Only.

[28] National Children’s Alliance (2021). Lighting the way: The broadening path of mental health services in CACs in the 21st century.

[29] Cohen JA, Mannarino AP (2015). Trauma-focused cognitive behavior therapy for traumatized children and families. Child Adolesc Psychiatric Clin North Am.

[30] Conners-Burrow NA, Tempel AB, Sigel BA, Church JK, Kramer TL, Worley KB (2012). The development of a systematic approach to mental health screening in Child Advocacy Centers. Children Youth Serv Rev.

[31] NCTSN Child Welfare Collaborative Group. Screening for mental health needs in the CAC. The National Child Traumatic Stress Network; 2017 Cited 2019 Feb 26. Available from: https://www.nctsn.org/sites/default/files/resources/fact-sheet/cac_screening_for_mental_health_needs_in_the_cac.pdf.

[32] Byrne KA, McGuier EA, Campbell KA, Shepard LD, Kolko DJ, Thorn B (2022). Implementation of a care process model for pediatric traumatic stress in Child Advocacy Centers: a mixed methods study. J Child Sex Abuse.

[33] Intermountain Healthcare (2020). Care Process Model: diagnosis and management of traumatic stress in pediatric patients.

[34] Shepard LD, Campbell KA, Byrne KA, Thorn B, Keeshin BR (2023). Screening for and responding to suicidality among youth presenting to a Children’s Advocacy Center (CAC). Child Maltreat.

[35] McGuier EA, Campbell KA, Byrne KA, Shepard LD, Keeshin BR. Traumatic stress symptoms and PTSD risk in children served by Children’s Advocacy Centers. Manuscript in preparation. 2023; 10.3389/fpsyt.2023.1202085 PMC10346839 37457766

[36] Rolon-Arroyo B, Oosterhoff B, Layne CM, Steinberg AM, Pynoos RS, Kaplow JB (2020). The UCLA PTSD Reaction Index for DSM-5 Brief Form: a screening tool for trauma-exposed youths. J Am Acad Child Adolesc Psychiatry.

[37] Mundt JC, Greist JH, Jefferson JW, Federico MA, Mann JJ, Posner KL (2013). Prediction of suicidal behavior in clinical research by lifetime suicidal ideation and behavior ascertained by the electronic Columbia-Suicide Severity Rating Scale. J Clin Psychiatry.

[38] Byington CL, Reynolds CC, Korgenski K, Sheng X, Valentine KJ, Nelson RE (2012). Costs and infant outcomes after implementation of a care process model for febrile infants. Pediatrics.

[39] Kaiser SV, Rodean J, Bekmezian A, Hall M, Shah SS, Mahant S (2018). Effectiveness of pediatric asthma pathways for hospitalized children: a multicenter, national analysis. J Pediatrics.

[40] Nkoy F, Fassl B, Stone B, Uchida DA, Johnson J, Reynolds C (2015). Improving pediatric asthma care and outcomes across multiple hospitals. Pediatrics.

[41] Panella M (2003). Reducing clinical variations with clinical pathways: do pathways work?. Int J Qual Health Care.

[42] Richardson KM, Fouquet SD, Kerns E, McCulloh RJ (2019). Impact of mobile device-based clinical decision support tool on guideline adherence and mental workload. Acad Pediatr.

[43] McGuier EA, Rothenberger SD, Campbell KA, Keeshin B, Weingart LR, Kolko DJ (2022). Team functioning and performance in Child Advocacy Center multidisciplinary teams. Child Maltreat.

[44] Ilgen DR, Hollenbeck JR, Johnson M, Jundt D (2005). Teams in organizations: from input-process-output models to IMOI models. Ann Rev Psychol.

[45] Kozlowski SWJ, Bell BS. Work groups and teams in organization. In: Borman WC, Ilgen DR, Klimoski RJ, editors. Handbook of Psychology (Vol 12): Industrial and Organizational Psychology. New York: Wiley-Blackwell; 2003 Cited 2020 Apr 10. 333–75. Available from: http://onlinelibrary.wiley.com/doi/abs/10.1002/9781118133880.hop212017.

[46] Rosen MA, Dietz AS. Team performance measurement. In: The Wiley Blackwell Handbook of the Psychology of Team Working and Collaborative Processes. Hoboken, NJ: John Wiley & Sons, Ltd; 2017. 479–502.

[47] Mathieu JE, Maynard MT, Rapp T, Gilson L (2008). Team effectiveness 1997–2007: a review of recent advancements and a glimpse into the future. J Manag.

[48] Courtright SH, Thurgood GR, Stewart GL, Pierotti AJ (2015). Structural interdependence in teams: an integrative framework and meta-analysis. J Appl Psychol.

[49] Kozlowski SWJ, Ilgen DR (2006). Enhancing the effectiveness of work groups and teams. Psychol Sci Public Interest.

[50] Kozlowski SWJ, Bell BS, Schmitt N, Highhouse S (2013). Work groups and teams in organizations: Review update. Handbook of Psychology (Vol 12): Industrial and Organizational Psychology.

[51] Van Der Vegt G, Emans B, Van De Vliert E (2000). Team members’ affective responses to patterns of intragroup interdependence and job complexity. J Manag.

[52] Bisbey T, Salas E. Team dynamics and processes in the workplace. In: Oxford Research Encyclopedia of Psychology. Oxford University Press; 2019. https://oxfordre.com/psychology/display/10.1093/acrefore/9780190236557.001.0001/acrefore-9780190236557-e-13.

[53] Weingart LR, Todorova G, Cronin MA (2010). Task conflict, problem-solving, and yielding: effects on cognition and performance in functionally diverse innovation teams. Negotiation Conflict Manag Res.

[54] Edmondson AC, Harvey JF (2018). Cross-boundary teaming for innovation: integrating research on teams and knowledge in organizations. Hum Resource Manag Rev.

[55] Cronin MA, Weingart LR (2007). Representational gaps, information processing, and conflict in functionally diverse teams. AMR.

[56] Hughes AM, Gregory ME, Joseph DL, Sonesh SC, Marlow SL, Lacerenza CN (2016). Saving lives: a meta-analysis of team training in healthcare. J Appl Psychol.

[57] Reiss-Brennan B, Brunisholz KD, Dredge C, Briot P, Grazier K, Wilcox A (2016). Association of integrated team-based care with health care quality, utilization, and cost. JAMA.

[58] Wilson KA (2005). Promoting health care safety through training high reliability teams. Qual Saf Health Care.

[59] Newman BS, Dannenfelser PL, Pendleton D (2005). Child abuse investigations: reasons for using Child Advocacy Centers and suggestions for improvement. Child Adolesc Soc Work J.

[60] Darlington Y, Feeney JA (2008). Collaboration between mental health and child protection services: professionals’ perceptions of best practice. Child Youth Serv Rev.

[61] Darlington Y, Feeney JA, Rixon K (2005). Interagency collaboration between child protection and mental health services: Practices, attitudes and barriers. Child Abuse Neglect.

[62] Ghan N (2016). Interagency collaboration in child abuse cases.

[63] Williams NJ, Beidas RS (2019). Annual research review: the state of implementation science in child psychology and psychiatry: a review and suggestions to advance the field. J Child Psychol Psychiatry.

[64] McGuier EA, Kolko DJ, Klem ML, Feldman J, Kinkler G, Diabes MA, et al. Team functioning and implementation of innovations in healthcare and human service settings: a systematic review protocol. Syst Rev. 2021;10(189):1-7. 10.1186/s13643-021-01747-w PMC8236140 34174962

[65] Edmondson AC, Bohmer RM, Pisano GP (2001). Disrupted routines: Team learning and new technology implementation in hospitals. Admin Sci Q.

[66] Wijnia L, Kunst EM, van Woerkom M, Poell RF (2016). Team learning and its association with the implementation of competence-based education. Teach Teach Educ.

[67] Lukas CV, Mohr D, Meterko M (2009). Team effectiveness and organizational context in the implementation of a clinical innovation. Qual Manag Health Care.

[68] Shortell SM, Marsteller JA, Lin M, Pearson ML, Wu SY, Mendel P (2004). The role of perceived team effectiveness in improving chronic illness care. Med Care.

[69] Graetz I, Reed M, Shortell SM, Rundall TG, Bellows J, Hsu J (2014). The association between EHRs and care coordination varies by team cohesion. Health Services Research..

[70] McGuier EA, Aarons GA, Byrne KA, Campbell KA, Keeshin B, Rothenberger SD (2023). Associations between teamwork and implementation outcomes in multidisciplinary cross-sector teams implementing a mental health screening and referral protocol. Implement Sci Commun.

[71] Landes SJ, McBain SA, Curran GM (2020). Reprint of: an introduction to effectiveness-implementation hybrid designs. Psychiatry Res.

[72] Curran GM, Landes SJ, McBain SA, Pyne JM, Smith JD, Fernandez ME, et al. Reflections on 10 years of effectiveness-implementation hybrid studies. Front Health Serv. 2022 ;2 Cited 2022 Dec 8. Available from: https://www.frontiersin.org/articles/10.3389/frhs.2022.1053496. 10.3389/frhs.2022.1053496 PMC10012680 36925811

[73] Bowen DJ, Kreuter M, Spring B, Cofta-Woerpel L, Linnan L, Weiner D (2009). How we design feasibility studies. Am J Prev Med.

[74] Eldridge SM, Lancaster GA, Campbell MJ, Thabane L, Hopewell S, Coleman CL (2016). Defining feasibility and pilot studies in preparation for randomised controlled trials: Development of a conceptual framework. PLOS One.

[75] Leon AC, Davis LL, Kraemer HC (2011). The role and interpretation of pilot studies in clinical research. J Psychiatric Res.

[76] Moore CG, Carter RE, Nietert PJ, Stewart PW (2011). Recommendations for planning pilot studies in clinical and translational research. Clin Transl Sci.

[77] Thabane L, Ma J, Chu R, Cheng J, Ismaila A, Rios LP (2010). A tutorial on pilot studies: the what, why and how. BMC Med Res Methodol.

[78] Brookman-Frazee L, Stahmer AC, Lewis K, Feder JD, Reed S (2012). Building a research-community collaborative to improve community care for infants and toddlers at-risk for autism spectrum disorders. J Commun Psychol.

[79] Drahota A, Meza RD, Brikho B, Naaf M, Estabillo JA, Gomez ED (2016). Community-academic partnerships: a systematic review of the state of the literature and recommendations for future research. Milbank Q.

[80] Lau AS, Rodriguez A, Bando L, Innes-Gomberg D, Brookman-Frazee L (2019). Research community collaboration in observational implementation research: Complementary motivations and concerns in engaging in the study of implementation as usual. Adm Policy Ment Health.

[81] Esmail L, Moore E, Rein A (2015). Evaluating patient and stakeholder engagement in research: moving from theory to practice. J Comp Effective Res.

[82] Ray KN, Miller E (2017). Strengthening stakeholder-engaged research and research on stakeholder engagement. J Comp Eff Res.

[83] Goodman MS, Ackermann N, Bowen DJ, Thompson V (2019). Content validation of a quantitative stakeholder engagement measure. J Commun Psychol.

[84] Guise JM, O’Haire C, McPheeters M, Most C, LaBrant L, Lee K (2013). A practice-based tool for engaging stakeholders in future research: a synthesis of current practices. J Clin Epidemiol.

[85] Luger TM, Hamilton AB, True G. Measuring community-engaged research contexts, processes, and outcomes: a mapping review. Milbank Q. 2020 .Cited 2020 May 20]; Available from: http://onlinelibrary.wiley.com/doi/abs/10.1111/1468-0009.12458. 10.1111/1468-0009.12458 PMC7296434 32428339

[86] Bartholomew LK, Parcel GS, Kok G (1998). Intervention mapping: a process for developing theory and evidence-based health education programs. Health Educ Behav.

[87] Belansky ES, Cutforth N, Chavez RA, Waters E, Bartlett-Horch K (2011). An adapted version of intervention mapping (AIM) is a tool for conducting community-based participatory research. Health Promot Pract.

[88] Dickson KS, Holt T, Arredondo E (2022). Applying Implementation Mapping to Expand a Care Coordination Program at a Federally Qualified Health Center. Front Public Health.

[89] Fernandez ME, ten Hoor GA, van Lieshout S, Rodriguez SA, Beidas RS, Parcel G, et al. Implementation mapping: Using intervention mapping to develop implementation strategies. Front Public Health. 2019 [Cited 2019 Jun 4];7. Available from: https://www.frontiersin.org/articles/10.3389/fpubh.2019.00158/abstract. 10.3389/fpubh.2019.00158 PMC6592155 31275915

[90] Highfield L, Valerio MA, Fernandez ME, Eldridge-Bartholomew LK. Development of an implementation intervention using intervention mapping to increase mammography among low income women. Front Public Health. 2018 [Cited 2019 Feb 25];6. Available from: https://www.ncbi.nlm.nih.gov/pmc/articles/PMC6212476/10.3389/fpubh.2018.00300 PMC6212476 30416992

[91] Pérez Jolles M, Fernández ME, Jacobs G, De Leon J, Myrick L, Aarons GA. Using Implementation Mapping to develop protocols supporting the implementation of a state policy on screening children for Adverse Childhood Experiences in a system of health centers in inland Southern California. Front Public Health. 2022;10. Cited 2022 Oct 27. Available from: https://www.frontiersin.org/articles/10.3389/fpubh.2022.876769. 10.3389/fpubh.2022.876769 PMC9459376 36091515

[92] Zwerver F, Schellart AJ, Anema JR, Rammeloo KC, van der Beek AJ (2011). Intervention mapping for the development of a strategy to implement the insurance medicine guidelines for depression. BMC Public Health.

[93] Salas E, DiazGranados D, Klein C, Burke CS, Stagl KC, Goodwin GF (2008). Does team training improve team performance? A meta-analysis. Hum Factors.

[94] Klein C, DiazGranados D, Salas E, Le H, Burke CS, Lyons R (2009). Does team building work?. Small Group Res.

[95] Shuffler ML, DiazGranados D, Salas E (2011). There’s a science for that: Team development interventions in organizations. Curr Dir Psychol Sci.

[96] Shuffler ML, Diazgranados D, Maynard MT, Salas E (2018). Developing, sustaining, and maximizing team effectiveness: An integrative, dynamic perspective of team development interventions. ANNALS.

[97] Lacerenza CN, Marlow SL, Tannenbaum SI, Salas E (2018). Team development interventions: Evidence-based approaches for improving teamwork. Am Psychol.

[98] Salas E, DiazGranados D, Weaver SJ, King H (2008). Does team training work? Principles for health care. Acad Emerg Med.

[99] Sheppard F, Williams M, Klein VR (2013). TeamSTEPPS and patient safety in healthcare. J Healthc Risk Manag.

[100] Weaver SJ, Dy SM, Rosen MA (2014). Team-training in healthcare: a narrative synthesis of the literature. BMJ Qual Saf.

[101] Smith-Jentsch KA, Cannon-Bowers JA, Tannenbaum SI, Salas E (2008). Guided team self-correction: Impacts on team mental models, processes, and effectiveness. Small Group Res.

[102] Miller CJ, Kim B, Silverman A, Bauer MS (2018). A systematic review of team-building interventions in non-acute healthcare settings. BMC Health Serv Res.

[103] Baker DP, Battles JB, King HB. New insights about team training from a decade of TeamSTEPPS. Perspectives on Safety. 2017 .Cited 2019 Jul 26; Available from: https://psnet.ahrq.gov/perspectives/perspective/218

[104] Capella J, Smith S, Philp A, Putnam T, Gilbert C, Fry W (2010). Teamwork training improves the clinical care of trauma patients. J Surg Educ.

[105] Clancy CM, Tornberg DN (2007). TeamSTEPPS: assuring optimal teamwork in clinical settings. Am J Med Qual.

[106] McGuier EA, Feldman J, Bay M, Ascione S, Tatum M, Salas E, et al. Improving teamwork in multidisciplinary cross-sector teams: Adaptation and pilot testing of a team training for Child Advocacy Center teams. In V. Byeon & A. Dopp (Chairs), Creating prepared, resilient, and equitable service systems: Multilevel approaches to preparing for and responding to disasters. Symposium presented at the annual convention of the Association for Behavioral and Cognitive Therapies; 2022 Nov; New York, NY.

[107] Kilbourne AM, Neumann MS, Pincus HA, Bauer MS, Stall R (2007). Implementing evidence-based interventions in health care: application of the replicating effective programs framework. Implement Sci.

[108] Kilbourne AM, Almirall D, Eisenberg D, Waxmonsky J, Goodrich DE, Fortney JC (2014). Protocol: Adaptive Implementation of Effective Programs Trial (ADEPT): cluster randomized SMART trial comparing a standard versus enhanced implementation strategy to improve outcomes of a mood disorders program. Implement Sci.

[109] Kilbourne AM, Abraham KM, Goodrich DE, Bowersox NW, Almirall D, Lai Z (2013). Cluster randomized adaptive implementation trial comparing a standard versus enhanced implementation intervention to improve uptake of an effective re-engagement program for patients with serious mental illness. Implementation Sci.

[110] Chan AW, Tetzlaff JM, Altman DG, Laupacis A, Gøtzsche PC, Krleža-Jerić K (2013). SPIRIT 2013 Statement: Defining Standard Protocol Items for Clinical Trials. Ann Intern Med.

[111] Chan AW, Tetzlaff JM, Gøtzsche PC, Altman DG, Mann H, Berlin JA (2013). SPIRIT 2013 explanation and elaboration: guidance for protocols of clinical trials. BMJ.

[112] Pinnock H, Barwick M, Carpenter CR, Eldridge S, Grandes G, Griffiths CJ (2017). Standards for Reporting Implementation Studies (StaRI) Statement. BMJ.

[113] Pinnock H, Barwick M, Carpenter CR, Eldridge S, Grandes G, Griffiths CJ (2017). Standards for Reporting Implementation Studies (StaRI): explanation and elaboration document. BMJ Open.

[114] Moher D, Hopewell S, Schulz KF, Montori V, Gotzsche PC, Devereaux PJ (2010). CONSORT 2010 Explanation and Elaboration: updated guidelines for reporting parallel group randomised trials. BMJ..

[115] Harris PA, Taylor R, Thielke R, Payne J, Gonzalez N, Conde JG (2009). Research electronic data capture (REDCap)—A metadata-driven methodology and workflow process for providing translational research informatics support. J Biomed Informatics.

[116] Harris PA, Taylor R, Minor BL, Elliott V, Fernandez M, O’Neal L (2019). The REDCap consortium: Building an international community of software platform partners. J Biomed Informatics.

[117] Finley EP, Huynh AK, Farmer MM, Bean-Mayberry B, Moin T, Oishi SM (2018). Periodic reflections: a method of guided discussions for documenting implementation phenomena. BMC Med Res Methodol.

[118] Proctor EK, Powell BJ, McMillen JC (2013). Implementation strategies: recommendations for specifying and reporting. Implementation Science..

[119] Bunger AC, Powell BJ, Robertson HA, MacDowell H, Birken SA, Shea C (2017). Tracking implementation strategies: a description of a practical approach and early findings. Health Research Policy and Systems..

[120] Orwin RG (2000). Assessing program fidelity in substance abuse health services research. Addiction..

[121] Weiner BJ, Lewis CC, Stanick C, Powell BJ, Dorsey CN, Clary AS (2017). Psychometric assessment of three newly developed implementation outcome measures. Implement Sci.

[122] Fernandez ME, Walker TJ, Weiner BJ, Calo WA, Liang S, Risendal B (2018). Developing measures to assess constructs from the Inner Setting domain of the Consolidated Framework for Implementation Research. Implement Sci.

[123] Aarons GA, Ehrhart MG, Farahnak LR (2014). The implementation leadership scale (ILS): development of a brief measure of unit level implementation leadership. Implement Sci.

[124] Aarons GA (2004). Mental health provider attitudes toward adoption of evidence-based practice: The Evidence-Based Practice Attitude Scale (EBPAS). Ment Health Serv Res.

[125] Ehrhart MG, Aarons GA, Farahnak LR. Going above and beyond for implementation: The development and validity testing of the Implementation Citizenship Behavior Scale (ICBS). Implement Sci. 2015 10(1) .Cited 2015 Jun 18. Available from: http://www.implementationscience.com/content/10/1/65. 10.1186/s13012-015-0255-8 PMC4465615 25948489

[126] Cronin MA, Bezrukova K, Weingart LR, Tinsley CH (2011). Subgroups within a team: The role of cognitive and affective integration. J Organiz Behav.

[127] Edmondson AC (1999). Psychological safety and learning behavior in work teams. Adminis Sci Q.

[128] Mattessich PW, Murray-Close M, Monsey BR. Collaboration: What makes it work. 2nd edition. Saint Paul, MN: Amherst H. Wilder Foundation; 2001. 10.

[129] Aubé C, Rousseau V (2005). Team goal commitment and team effectiveness: the role of task interdependence and supportive behaviors. Group Dynamics.

[130] Kearney E, Gebert D, Voelpel SC (2009). When and how diversity benefits teams: tThe importance of team members’ need for cognition. Acad Manag J.

[131] Gittell JH, Fairfield KM, Bierbaum B, Head W, Jackson R, Kelly M (2019). Impact of relational coordination on quality of care, postoperative pain and functioning, and length of stay: a nine-hospital study of surgical patients. Med Care.

[132] Mathieu JE, Luciano MM, D’Innocenzo L, Klock EA, LePine JA (2020). The development and construct validity of a team processes survey measure. Organ Res Methods.

[133] Schippers MC, Den Hartog DN, Koopman PL (2007). Reflexivity in teams: a measure and correlates. Applied Psychology..

[134] Battles J, King HB (2010). TeamSTEPPS® Teamwork Perceptions Questionnaire Manual.

[135] Eby LT, Meade AW, Parisi AG, Douthitt SS (1999). The development of an individual-level teamwork expectations measure and the application of a within-group agreement statistic to assess shared expectations for teamwork. Organizational Res Methods..

[136] Braun V, Clarke V (2006). Using thematic analysis in psychology. Qual Res Psychol.

[137] Gale RC, Wu J, Erhardt T, Bounthavong M, Reardon CM, Damschroder LJ (2019). Comparison of rapid vs in-depth qualitative analytic methods from a process evaluation of academic detailing in the Veterans Health Administration. Implement Sci.

[138] Taylor B, Henshall C, Kenyon S, Litchfield I, Greenfield S (2018). Can rapid approaches to qualitative analysis deliver timely, valid findings to clinical leaders? A mixed methods study comparing rapid and thematic analysis. BMJ Open.

[139] Barusch A, Gringeri C, George M (2011). Rigor in qualitative social work research: a review of strategies used in published articles. Soc Work Res.

[140] Birt L, Scott S, Cavers D, Campbell C, Walter F. Member checking: A tool to enhance trustworthiness or merely a nod to validation? Qualitative Health Research. 2016 [Cited 2020 Jun 8]; Available from: https://journals.sagepub.com/doi/10.1177/1049732316654870 . 10.1177/1049732316654870 27340178

[141] Carlson JA (2010). Avoiding traps in member checking. Qual Rep.

[142] Aarons GA, Fettes DL, Sommerfeld DH, Palinkas LA (2012). Mixed methods for implementation research: application to evidence-based practice implementation and staff turnover in community-based organizations providing child welfare services. Child Maltreat.

[143] Palinkas LA, Aarons GA, Horwitz S, Chamberlain P, Hurlburt M, Landsverk J (2011). Mixed method designs in implementation research. Adm Policy Ment Health.

[144] Rehnborg SJ, Carpluk W, French V, Lin S, Repp D, Seals C (2009). Outcome Measurement System: Final report for the Children’s Advocacy Centers of Texas, Inc.

[145] Bernal JL, Cummins S, Gasparrini A (2017). Interrupted time series regression for the evaluation of public health interventions: a tutorial. Int J Epidemiol.

[146] Kontopantelis E, Doran T, Springate DA, Buchan I, Reeves D (2015). Regression based quasi-experimental approach when randomisation is not an option: interrupted time series analysis. BMJ.

[147] International Committee of Medical Journal Editors. Recommendations for the conduct, reporting, editing, and publication of scholarly work in medical journals. 2021 [cited 2021 Dec 21]. Available from: http://www.icmje.org/icmje-recommendations.pdf. 25558501

[148] Lewis CC, Klasnja P, Powell BJ, Lyon AR, Tuzzio L, Jones S, et al. From classification to causality: Advancing understanding of mechanisms of change in implementation science. Front Public Health. 2018; 6 [cited 2018 Oct 12]. Available from: https://www.frontiersin.org/articles/10.3389/fpubh.2018.00136/full?&utm_source=Email_to_authors_&utm_medium=Email&utm_content=T1_11.5e1_author&utm_campaign=Email_publication&field=&journalName=Frontiers_in_Public_Health&id=336504. 10.3389/fpubh.2018.00136 PMC5949843 29868544

[149] Lewis CC, Boyd MR, Walsh-Bailey C, Lyon AR, Beidas R, Mittman B (2020). A systematic review of empirical studies examining mechanisms of implementation in health. Implement Sci.

[150] Lewis CC, Klasnja P, Lyon AR, Powell BJ, Lengnick-Hall R, Buchanan G (2022). The mechanics of implementation strategies and measures: advancing the study of implementation mechanisms. Implement Sci Commun.

